# Colorectal Cancer—One Disease, Two Fires: Distinct Inflammatory Landscapes in Colon and Rectal Cancer

**DOI:** 10.3390/diagnostics15182387

**Published:** 2025-09-19

**Authors:** Catalin Vladut Ionut Feier, Florin Grama, Georgiana Viorica Moise, Razvan Constantin Vonica, Vasile Gaborean, Alaviana Monique Faur, Vladut Iosif Rus, Calin Muntean

**Affiliations:** 1 Abdominal Surgery and Phlebology Research Center, “Victor Babeș” University of Medicine and Pharmacy Timişoara, 300041 Timişoara, Romania; catalin.feier@umft.ro; 2 First Surgery Clinic, “Pius Brinzeu” Clinical Emergency Hospital, 300723 Timişoara, Romania; 3 Faculty of Medicine, “Carol Davila” University of Medicine and Pharmacy, 020021 Bucharest, Romania; 4 Clinical General Surgery Department, Colțea Clinical Hospital, 030171 Bucharest, Romania; 5 Department of Doctoral Studies, “Victor Babeş” University of Medicine and Pharmacy Timişoara, 300041 Timişoara, Romania; georgiana.moise@umft.ro; 6 Medical Oncology, “Pius Brinzeu” Clinical Emergency Hospital, 300723 Timişoara, Romania; 7 Preclinical Department, Discipline of Physiology, Faculty of Medicine, “Lucian Blaga” University of Sibiu, 550169 Sibiu, Romania; 8 Department of Oncology, “Elysee Hospital”, 510040 Alba Iulia, Romania; 9 Thoracic Surgery Research Center, “Victor Babeş” University of Medicine and Pharmacy Timişoara, 300041 Timişoara, Romania; vasile.gaborean@umft.ro; 10 Department of Surgical Semiology, Faculty of Medicine, “Victor Babeş” University of Medicine and Pharmacy Timişoara, 300041 Timişoara, Romania; 11 Faculty of Medicine, “Victor Babeş” University of Medicine and Pharmacy Timişoara, 300041 Timişoara, Romania; alaviana.faur@student.umft.ro (A.M.F.); iosif.rus@student.umft.ro (V.I.R.); 12 Medical Informatics and Biostatistics, Department III-Functional Sciences, “Victor Babeş” University of Medicine and Pharmacy Timişoara, 300041 Timişoara, Romania; cmuntean@umft.ro

**Keywords:** colon cancer, rectal cancer, systemic inflammation, inflammatory biomarkers, neutrophil-to-lymphocyte ratio, platelet-to-lymphocyte ratio, systemic immune-inflammation index, elective surgery, emergency surgery

## Abstract

**Background:** Systemic inflammatory indices are increasingly used to predict prognosis in colorectal cancer (CRC), yet direct comparisons between colon cancer (CC) and rectal cancer (RC) remain limited. **Methods:** We conducted a retrospective matched-cohort study including 296 patients (148 with CC and 148 with RC) surgically treated between January 2018 and December 2024. Patients were matched by tumor stage, sex, and age (±3 years). Preoperative blood samples were used to calculate several inflammatory markers, including Neutrophil-to-Lymphocyte Ratio (NLR), Platelet-to-Lymphocyte Ratio (PLR), Monocyte-to-Lymphocyte Ratio (MLR), Systemic Inflammation Response Index (SIRI), Systemic Immune-Inflammation Index (SII), and Aggregate Index of Systemic Inflammation (AISI). Subgroup analyses were performed based on the Charlson Comorbidity Index (>3 vs. ≤3), surgical context (elective vs. emergency), and tumor stage (T1–T2 vs. T3–T4). **Results:** Colon cancer patients exhibited significantly higher levels of systemic inflammation compared to those with rectal cancer, with notable differences in NLR (3.99 vs. 2.84, *p* < 0.001), PLR (219.8 vs. 163.3, *p* < 0.001), SIRI (3.7 vs. 1.91, *p* = 0.004), SII (1533.8 vs. 847.8, *p* < 0.001), and AISI (1714.7 vs. 593.6, *p* = 0.009). These differences remained statistically significant in key subgroups. In elective surgeries, CC patients had elevated PLR (*p* < 0.001), SIRI (*p* = 0.003), SII (*p* < 0.001), and AISI (*p* = 0.013). Among patients with advanced tumors (T3–T4), CC was associated with higher SII (*p* < 0.001), AISI (*p* = 0.008), PLR (*p* < 0.001), and SIRI (*p* = 0.004). For those with a Charlson index > 3, CC patients showed significantly higher PLR (*p* < 0.001), NLR (*p* < 0.001) and SIRI (*p* = 0.001). **Conclusions:** colon cancer presents with a markedly stronger systemic inflammatory response than rectal cancer, particularly in patients with advanced disease, elective surgical treatment, and higher comorbidity burden. These findings suggest that indices such as SIRI, SII, and PLR may serve as valuable stratification tools beyond tumor location in CRC.

## 1. Introduction

Colorectal cancer (CRC) has emerged as a significant public health concern nowadays, currently ranking as the third most commonly diagnosed cancer worldwide and the second leading cause of cancer-related mortality globally [1]. According to GLOBOCAN 2022 data, more than 1.9 million new CRC cases and approximately 935,000 CRC-related deaths were reported worldwide, with rectal cancer being responsible for a considerable share—over 310,000 deaths representing one-third of all CRC cases [2]. While countries with well-developed healthcare systems (USA, Germany, and France) have made significant reductions in CRC mortality through organized screening programs and early detection initiatives, many regions in Eastern and Central Europe continue to face serious challenges in diagnosing early cases [1,3,4]. These persistent disparities stem from structural healthcare limitations, inadequate screening, and a very low public awareness, altogether leading to a high proportion of cases being addressed at advanced stages. Even if colon cancer (CC) and rectal cancer (RC) are often regarded as one pathology within the medical community, treatment approaches have increasingly diverged, reflecting differing disease courses and outcomes [5].

These regional patterns are reflected within the Romanian healthcare system as well. Studies conducted in this country reveal that CC localizations, particularly the ones located in the right and left colon, account for more than 73% of all CRC cases. Among these, a slight predominance of right-sided CC was observed in female patients [6]. Colonic tumors are frequently associated with older age groups (>70 years) and carry an increased risk of recurrence, especially in proximal colon cancers [7,8].

In a study performed in Timisoara, Romania, RC represents a third of CRC cancer cases. The incidence of this pathology was notably higher in patients aged 50–69 years. Unlike CC, RC were more commonly associated with the male gender, and did not show statistically significant age-related differences in tumor aggressiveness [8].

In spite of significant advancements in surgical techniques, staging systems, and adjuvant therapies, the prognosis of CC remains strongly dependent on tumor stage. Five-year overall survival rates vary considerably, reaching almost 90% for stage I and declining to almost 65% for stage III [5,9]. Prognosis for RC has notably improved with the adoption of neoadjuvant chemoradiotherapy (nCRT), total mesorectal excision (TME), and multidisciplinary treatment approaches. However, outcomes remain variable, with five-year overall survival rates of 85% in stage I and 55% in stage III. Regarding the stage IV patients, both CC and RC face markedly poor survival, often below 15%, largely due to limited responsiveness to conventional treatment regimens [9,10].

Among the most accessible and reproducible prognostic tools in CRC are inflammation-based biomarkers derived from standard blood counts, including the Neutrophil-to-Lymphocyte Ratio (NLR), Platelet-to-Lymphocyte Ratio (PLR), Systemic Immune-Inflammation Index (SII), Systemic Inflammation Response Index (SIRI), and Aggregate Index of Systemic Inflammation (AISI). Elevated pre-treatment levels of these markers have consistently been associated with poor tumor regression, reduced disease-free survival (DFS), and lower overall survival (OS), with reported hazard ratios ranging from 1.5 to 3.2 in multivariate models. For instance, NLR represents the balance between pro-tumorigenic inflammation and adaptive immunity [11,12,13]. Studies have shown that an NLR ≥ 2.3 has been linked to significantly inferior 5-year OS, while SII values above 700 have predicted poor pathological response and increased recurrence risk [14]. The thrombosis-inflammation interactions are reflected by PLR. Values over 150–200 indicate platelet activation leading to tumor cell adhesion and metastatic potential through TGF-β and PDGF release [11,15]. More complex markers, such as SII, provide a more detailed inflammation assessment. Studies have shown that values over 700 indicate a proinflammatory, pro-thrombotic state with impaired immune surveillance, correlated with poor neoadjuvant response and a significant decrease in survival [16,17]. SIRI also belongs to this group, as it highlights inflammation driven by monocytes. Values exceeding 2 have been linked to activation of tumor-associated macrophages, which contribute to angiogenesis and immunosuppression by secreting IL-10, TGF-β, and VEGF [18]. AISI is the most inclusive index, combining all key inflammatory factors. Levels above 500–1000 reflect severe systemic inflammation, which fosters an environment favorable for tumor growth [19,20]. High pre-treatment values of this marker are consistently linked to poorer tumor response, shorter disease-free survival, and decreased overall survival, with hazard ratios ranging from 1.5 to 3.2 in multivariate analyses [16,17,20].

However, despite growing evidence supporting their prognostic value, the overwhelming majority of studies continue to treat CRC as a unified entity, overlooking the distinct biological and clinical profiles of CC and RC. Few publications stratify results by tumor site, and even fewer directly compare inflammatory marker behavior between CC and RC. For example, a large prospective study by Ose et al. [21] showed that preoperative CRP and adhesion molecule levels had divergent prognostic implications in colon versus rectal tumors—yet no inflammatory index like NLR or SIRI was analyzed separately by site [13,21]. While isolated studies have explored systemic inflammation in RC or CC alone [15,17,22,23], the literature lacks head-to-head evaluations of inflammation-based markers between these two tumor types. Given their differing embryologic origins, immune microenvironments, and treatment paradigms, understanding whether systemic inflammatory responses diverge between CC and RC could have meaningful implications for personalized risk stratification and therapeutic targeting.

The primary objective of this study was to determine whether CC and RC elicit distinct systemic inflammatory responses before surgery by directly comparing standard inflammatory indices between the two. By selecting matched patient groups with similar age, gender, and tumor stage, we aimed to isolate the effect of tumor location itself—colon vs. rectum—on the immune-inflammatory profile. This approach helps clarify whether CC and RC should be evaluated as distinct entities when using inflammation-based prognostic markers.

## 2. Materials and Methods

In order to conduct this study, data were analyzed from the medical records of patients diagnosed with CC and RC, who underwent surgical treatment and were monitored at the medical oncology outpatient clinic of the “Pius Brînzeu” County Emergency Clinical Hospital in Timișoara, Romania. Data collection for this retrospective study included patients diagnosed and surgically treated for CC or RC between 1 January 2018 and 1 December 2024, at the “Pius Brînzeu” Clinical Emergency Hospital in Timișoara, Romania. Initially, 236 patients with CC and 174 patients with RC were identified. In order to select appropriate cases, inclusion and exclusion criteria were established. Only patients who had a confirmed postoperative histopathological diagnosis of colon adenocarcinoma or rectal adenocarcinoma were taken into consideration. Two patient cohorts, with the same size (148 patients in each cohort) and matching key characteristics, were selected. Specifically, patients in both groups were matched for disease stage (I–IV), age (±3 years), and gender, given the known fact that age, gender, and cancer stage have a significant influence on inflammatory status [8,9,20].

**Figure 1:**
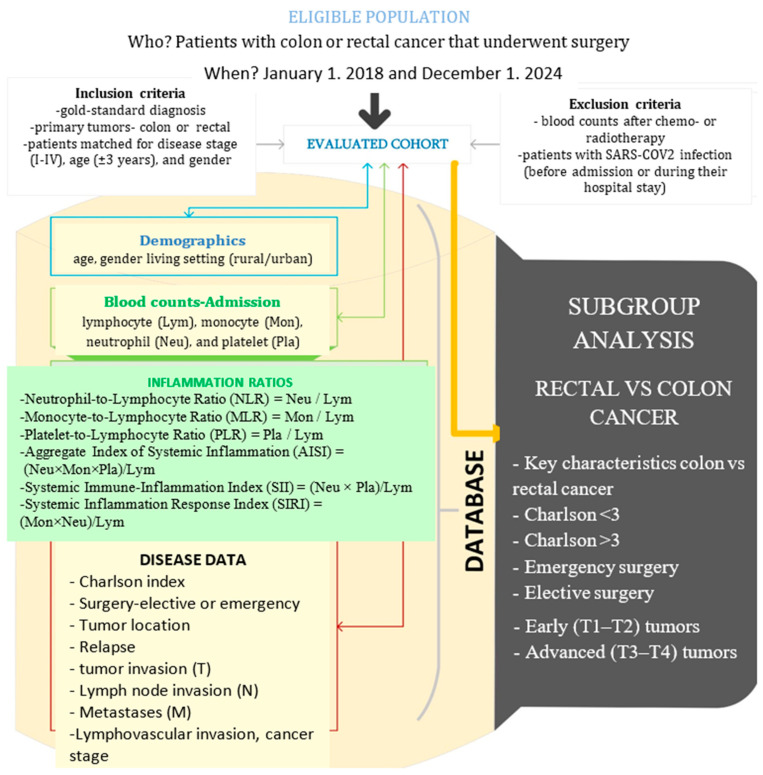
The setting, raw, and derived data in our study (drawn with Microsoft Visio, v. 16.0, 2019, Microsoft Corporation, Redmond, WA, USA).

Figure 1 presents the flow of the applied methods. The histopathological examination was the gold standard diagnosis in our study. Medical charts of patients comprised the source of raw data.

Given that the study timeframe coincided with the COVID-19 pandemic, individuals with confirmed SARS-CoV-2 infection either before admission or during hospitalization were excluded, due to the virus’s known impact on systemic inflammation [24,25,26].

Due to the presence of both CC and RC patients, the timing of the blood sample collection was essential. The known impact of chemo-radiotherapy on systemic inflammatory markers [27] was taken into consideration. Thus, only patients with available pre-treatment blood tests—either prior to emergency surgery or, in elective cases, before starting neoadjuvant therapy—were included. In the colon cancer group, only preoperative blood samples were considered, and patients who had received any form of adjuvant chemotherapy were excluded from the analysis.

To assess comorbidities, the Charlson Comorbidity Index was employed. Data on recurrence were also recorded. Histopathological parameters of the resected specimens, including tumor invasion (T), lymph node involvement (N), presence of metastases (M), and lymphatic invasion, were analyzed along with the tumor stage.

The study was conducted in accordance with the Declaration of Helsinki and approved by the Ethics Committee of “Pius Brînzeu” Clinical Emergency Hospital, Timișoara, Romania (Approval No. 538/8 April 2025).

### Statistical Analyses

All statistical analyses were carried out using IBM SPSS Statistics version 25.0 for Windows (IBM Corp., Armonk, NY, USA). The Shapiro–Wilk test was employed to assess the normality of numerical data distribution. Continuous data were described using means and standard deviations or medians and interquartile ranges, depending on distribution. Categorical variables were summarized as absolute frequencies and percentages. To compare two independent groups, the Student’s *t*-test was used for normally distributed data, while the Mann–Whitney U test was applied for non-normally distributed variables. Group differences in categorical data were examined using either the Chi-square test or Fisher’s exact test, depending on cell frequencies. A two-tailed *p*-value < 0.05 was considered statistically significant for all analyses.

## 3. Results

In order to conduct this study, data from 296 patients aged between 31 and 82 years were collected. Only patients who were followed up at the outpatient oncology department of the “Pius Brînzeu” County Emergency Clinical Hospital in Timișoara were taken into consideration.

### 3.1. Key Information

Patients with CC were less likely to come from rural areas and had a lower proportion with a Charlson index > 3, as well as nearly four times fewer relapse cases. However, they presented more frequently with lymphatic invasion and a higher proportion of tumors staged as T4.

The characteristics of the 2 groups are presented in Table 1.

We proceeded to analyze the variation of inflammatory markers between the two groups of patients. After the statistical analysis, the results revealed statistically significant differences in the majority of them, as seen in Table 2. The only notable exceptions that did not present significant differences were the monocyte count and the MLR.

### 3.2. Impact of Comorbidities

Given the observed differences in the proportion of patients with a Charlson index > 3, we further investigated the presence of significant distinctions between CC and RC patients from multiple perspectives.

Initially, we focused on patients who had a Charlson index < 3. Among the 296 patients, 133 (44.93%) had a Charlson index below 3, of which 78 (52.7%) were CC patients, and 55 (37.4.%) were RC patients. No significant differences were observed between the 2 groups regarding the proportion of male patients (*p* = 0.215), urban residence (*p* = 0.176), T stage (*p* = 0.288), N stage (*p* = 0.262), cancer stage (*p* = 0.976), or age (*p* = 0.327).

In this subset of patients without any associated comorbidities (Charlson index < 3), no significant differences were observed in the variation of inflammatory markers between the two groups. The results are presented in Table 3.

The situation differed in patients presenting with at least one comorbidity associated with cancer. Considering patients with a Charlson index > 3, 163 individuals (55.07%) fell into this category, of whom 71 (43.55%) were diagnosed with CC and 92 (56.45%) with RC.

No significant differences were identified between the two pathologies regarding the proportion of male patients (*p* = 0.286), N stage (*p* = 0.134), overall stage (*p* = 0.857), or age (*p* = 0.153). However, significant differences were identified between the proportions of patients from urban areas, with 50 (71.4%) in one group versus 43 (46.7%) in the other (*p* = 0.001).

At the time of evaluating the variation of inflammatory markers, statistical analysis revealed significant differences between the two groups for most markers, with the exception of monocyte count and the MLR. The results are presented in Table 4.

### 3.3. Emergency vs. Elective Surgery

Regarding the type of surgery performed, out of the 296 patients, 78 (26.4%) underwent emergency surgery, while the remaining 218 (73.6%) underwent elective procedures. No significant differences were observed in the proportion of patients undergoing emergency surgery between those with CC and RC (*p* = 0.117). Specifically, 34 patients (43.58%) had CC and 44 patients (56.41%) had RC.

Initially, patients who underwent emergency surgery for the treatment of CC or RC were analyzed. Patients with CC and RC requiring emergency surgery did not exhibit significant differences in terms of the proportion of male patients (*p* = 0.819), age (*p* = 0.669), N stage (*p* = 0.207), or overall disease stage (*p* = 0.104).

These patients showed significant differences between proportions regarding the following variables:

- Urban residence (22 (53.7%) vs. 12 (32.4%), *p* = 0.048)
- T stage (*p* = 0.022):
  ○ T2 (2 (5.9%) vs. 0)
  ○ T3 (16 (47.1%) vs. 33 (75%))
  ○ T4 (16 (47.1%) vs. 11 (25%))

Although only two aspects showed significant differences between patients with CC and RC who underwent emergency surgery, the variation in inflammatory markers between the two pathologies did not differ significantly within this patient subgroup. The variation and differences between the two groups are presented in Table 5.

The only parameter that showed a statistically significant difference in mean values between CC and RC was the monocyte-to-lymphocyte ratio (MLR), with values of 0.3 ± 0.21 versus 0.45 ± 0.3, respectively (*p* = 0.037).

Among patients who underwent elective surgery, 114 (52.3%) had CC, and the remaining 104 (47.7%) had RC. No significant differences were observed in the proportion of patients undergoing elective surgery between the CC and RC groups (*p* = 0.117). Patients with CC and RC who required surgical intervention did not show significant differences regarding the proportion of male patients (*p* = 0.431), age (*p* = 0.077), N stage (*p* = 0.276), or overall disease stage (*p* = 0.696).

These patients showed significant differences in the proportions regarding:

- Urban residence (78 (68.4%) vs. 54 (51.9%), *p* = 0.009)
- T stage *p* = 0.004:
  ○ T1 (4 (3.5%) vs. 2 (1.9%))
  ○ T2 (10 (8.8%) vs. 20 (19.2%))
  ○ T3 (64 (56.1%) vs. 68 (65.4%))
  ○ T4 (36 (31.6%) vs. 11 (13.5%))

In contrast to patients undergoing emergency surgery, where no significant differences were identified in the variation of inflammatory markers, patients operated on electively showed statistically significant changes in the majority of evaluated parameters between CC and RC. The results are presented in Table 6.

### 3.4. Evaluating Early (T1–T2) vs. Advanced (T3–T4) Tumors

Given that significant differences in T stage variation were observed between CC and RC patients within each group, we decided to compare two samples: those presenting with T1–T2 stage and those with T3–T4 stage.

Among the patients, 38 (12.83%) presented with T1–T2 stage, of whom 16 (42.1%) had CC and 22 (57.9%) had RC. No significant differences were identified between the two proportions (*p* = 0.385).

In this limited group, no significant differences were observed between the two pathologies regarding the proportion of male patients (*p* = 0.187), urban residence (*p* = 0.309), N stage (*p* = 0.143), overall disease stage (*p* = 0.148), presence of Charlson index >3 (*p* = 0.188), or age (*p* = 0.218).

No significant differences were identified regarding the inflammatory ratios investigated; however, differences in blood cell counts were observed. The results are presented in Table 7.

Among the patients, 258 (87.16%) presented with T3–T4 stage, of whom 132 (51.16%) had CC and 126 (48.84%) had RC. No significant differences were identified between the two proportions (*p* = 0.455).

No significant differences were observed between the two pathologies regarding the proportion of male patients (*p* = 0.796), N stage (*p* = 0.406), overall disease stage (*p* = 0.795), or age (*p* = 0.378). However, significant differences were identified in the proportions of patients from urban areas (88 (66.7%) vs. 61 (48.4%), *p* = 0.004), although no significant difference was found in the presence of a Charlson index >3 (64 (48.5%) vs. 78 (62.4%), *p* = 0.188).

In these patients, significant differences were observed in the majority of the investigated inflammatory parameters, with the exception of monocyte count and MLR. The results are presented in Table 8.

**Figure 2:**
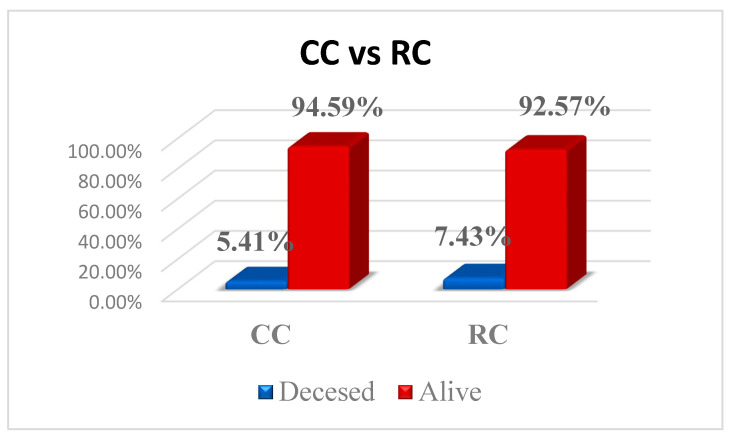
Deceased patients CC vs. RC.

Of the 296 patients, 19 died (8 for CC and 11 for RC, with no significant differences between, *p* = 0.643); however, the exact time interval between the surgical intervention and death is unknown. The proportion of these patients according to the type of pathology is presented in Figure 2.

## 4. Discussion

Recently, the management and treatment of CC and RC have made serious advancements, specifically with the aid of multimodal therapeutic approaches and the identification of new prognostic markers. Treatment strategies include surgeries, chemotherapy, and nCRT, all of them improving significantly the clinical outcomes [5,8,10]. However, patients’ prognosis remains highly variable, dependent on the stage and individual biological characteristics [9,10,28]. Therefore, the evaluation of accessible clinical and biological markers becomes essential for more precise risk stratification and personalized treatment, aiming to optimize therapeutic results. This context underscores the ongoing need for research and monitoring of these markers in clinical practice to adapt therapeutic strategies to the specific needs of each patient.

In our study, the proportion of patients from rural areas was significantly higher in RC (50.7% vs. 32.4% in colon cancer; *p* = 0.002), which may affect access to screening and early treatment. This finding aligns with other studies that reported increased incidence of advanced colorectal cancers in rural populations, likely due to limited preventive and diagnostic healthcare services [1,29,30]. Additionally, a Charlson score > 3, indicating greater comorbidity burden, was more frequent in RC (62.6% vs. 47.3%, *p* = 0.010). The greater Charlson score in RC patients likely reflects a later presentation in older individuals, often from rural areas with limited access to early medical care. These patients tend to accumulate more chronic illnesses over time, which contributes to the higher comorbidity burden observed at diagnosis [31,32].

Interestingly, CC patients had about four times fewer recurrences (4.1% vs. 15.5%, *p* = 0.001) compared to RC patients, likely due to the distinct biological and therapeutic characteristics of rectal tumors, where technical challenges of total mesorectal excision and more aggressive local behavior increase local recurrence risk [21,33].

When analyzing the inflammatory parameters, our analysis revealed significant differences in nearly all parameters assessed, with the exception of monocyte count and MLR. These results highlight the possibility that monocytes play a more intricate role in colorectal carcinogenesis than what can be inferred from peripheral blood values alone. Within the tumor microenvironment, monocytes can differentiate into distinct subsets with varying immunological functions, potentially exerting different effects in CC compared to RC. However, these intratumoral dynamics are not easily reflected in systemic markers such as absolute monocyte counts or MLR [34]. Lymphocyte levels were significantly lower in CC (1697 ± 681) compared to RC (2048 ± 830; *p* < 0.001), suggesting a state of lymphopenia that may reflect impaired immune surveillance and facilitate tumor progression. This aligns with the work of Coussens, who emphasized the critical role of adaptive immunity in tumor control mechanisms [35].

Inflammatory ratios such as NLR (*p* < 0.001), PLR (*p* < 0.001), and SII (*p* < 0.001) were significantly elevated in CC, indicating a more pronounced systemic inflammatory response. These markers have consistently been linked with poor outcomes in CRC, as demonstrated in large-scale studies [11,14]. Among them, NLR has emerged as a robust predictor of tumor progression and survival, and the differences observed between CC and RC may reflect underlying disparities in tumor–microenvironment interactions [11]. Composite indices such as SII (*p* <0.001) and SIRI (*p* = 0.004) were also markedly higher in CC, further supporting a heightened inflammatory state. These findings underscore a distinct systemic inflammatory profile in CC, which may reflect underlying differences in tumor immunogenicity, stromal composition, and microbiota-driven immune modulation compared to RC [36,37].

### 4.1. Impact of Comorbidities

Our data reveal a clear stratification of systemic inflammatory burden according to comorbidity status, as assessed by the Charlson Comorbidity Index in both CC and RC. Among patients with low comorbidity (CCI ≤ 3), the differences in inflammatory markers between CC and RC were relatively modest and did not reach statistical significance, except for lymphocyte counts, which remained significantly lower in CC (*p* = 0.022). However, when speaking about the patients with important comorbidities, the differences became significant for most of the investigated parameters (neutrophil count, NLR, PLR, AISI, SIRI, SII) with higher values in CC patients. This suggests that, in the absence of additional comorbidity-related inflammatory stimuli, colonic and rectal tumors may exhibit more comparable systemic immune profiles. Notably, these findings align with recent observations that comorbidities can amplify baseline inflammatory markers, thereby confounding their tumor-specific prognostic value in CRC populations [31,38,39].

Moreover, a more pronounced inflammatory activation observed in CC may be due to the distinct molecular immunologic and microbial characteristics. Particularly, right-sided colon cancers are frequently classified under CMS1 (microsatellite instability–high, immune-active), a subtype marked by dense immune infiltration and elevated systemic inflammation [40,41]. This intrinsic immune activation is often further amplified by comorbidity-driven inflammation.

### 4.2. Elective vs. Emergency Surgery

Patients undergoing elective surgery exhibited markedly greater differences between CC and RC across multiple systemic inflammatory markers—including NLR (*p*  <  0.001), PLR, SII, and AISI—than those treated in emergency settings, where these differences were attenuated (e.g., NLR *p*  =  0.49).

It is a well-known fact that emergency interventions involve acute clinical situations, often complicated by conditions such as perforations, severe infections, ischemia, or peritonitis, that reveal a pronounced and nonspecific inflammatory response. This widespread inflammation leads to a fast and significant increase in hematological inflammatory parameters (NLR, PLR, SII, SIRI, AISI), regardless of the tumor’s etiology or location [17,20]. Thus, acute systemic inflammation “masks” the inherent differences between CC and RC, reducing the predictive and discriminatory value of these biomarkers in the context of emergency surgeries.

On the other hand, patients who underwent elective surgeries are generally in a more stable clinical condition, allowing for a more accurate assessment of tumor-specific inflammation and evaluation of the differences between CC and RC. Moreover, elective surgeries enable better planning, management of comorbidities, and administration of adjuvant treatments, which may explain the more pronounced differences observed in inflammatory markers, especially in CC [20,33].

Therefore, evaluating inflammatory markers in the elective setting can serve as a valid tool for prognostic stratification and personalized management. In contrast, during emergency interventions, these markers tend to be less specific due to the acute systemic inflammatory response, reducing their predictive and discriminatory value. This distinction highlights the importance of context when interpreting inflammatory biomarkers in colorectal cancer patients.

### 4.3. Early (T1–T2) vs. Advanced (T3–T4) Tumors

When evaluating CC and RC depending on the characteristics of tumor invasion, the investigated markers diverged more significantly between CC and RC in advanced tumors (T3–T4). In the early tumors, no significant differences were observed between the two groups in the investigated ratios (NLR, PLR, AISI, SIRI, SII). However, things presented a completely different perspective when we evaluated the results for the T3–T4 group. In this subset, key biomarkers (NLR, PLR, SII) presented extremely significant differences with a *p* < 0.001, while AISI and SIRI presented strong differences as well in patients with CC cancer. This differential response may reflect the complex tumor microenvironment of CC, which becomes progressively more immunologically reactive as the disease advances. Elevated systemic inflammation is driven by chronic release of proinflammatory cytokines (e.g., IL-6, TNF-α), recruitment of neutrophils and macrophages, and expansion of Th1/Th17 responses—features linked to tumor progression and immune dysregulation in colon cancer [42,43].

Moreover, increased platelet activity in advanced CC, indicated by elevated PLR, contributes to tumor angiogenesis and metastatic potential [11,15]. The predominance of CMS1 molecular subtype in right-sided CC, characterized by immune activation and high microsatellite instability (MSI), further explains the stronger systemic inflammatory profile compared to RC [40,41]. In contrast, early-stage tumors (T1–T2) displayed minimal inflammatory differences between CC and RC, consistent with a more immunologically quiescent microenvironment and a favorable prognosis [44].

As seen throughout all our studies, there were significant differences between CC and RC regarding the inflammatory status. Even if there is a low number of studies that approach this subject [45,46,47], there are few explanations, such as distinct molecular, immunological, and microbial characteristics that support the differences between CC and RC. Colon tumors, particularly those in the right colon, often correspond to the CMS1 molecular subtype characterized by MSI and a strong T-cell immune infiltration. This immune activity is mediated by a proinflammatory and immunoactive tumor microenvironment [40,41]. Such intense immune engagement leads to a state of chronic inflammation, both locally and systemically, which is reflected in increased levels of inflammatory markers such as NLR, PLR, and SII.

Moreover, these distinct systemic inflammatory marker profiles may be partly explained by the microbiota of the right colon that contributes significantly to this inflammatory response. Numerous studies have found that *Fusobacterium nucleatum* is frequently enriched in colon cancer tissues, especially in the right colon, where it promotes production of proinflammatory cytokines like IL-6 and TNF-α. These cytokines recruit neutrophils and activate platelets, amplifying systemic inflammation [47,48]. *F. nucleatum* enhances colorectal tumorigenesis through mechanisms, including the activation of NF-κB and TLR-mediated inflammatory pathways, contributing to a chronically inflamed tumor microenvironment that favors tumor progression and aggressiveness [49]. High intratumoral loads of *F. nucleatum* have also been associated with worse clinical outcomes and increased metastasis in colorectal cancer patients [50].

In contrast, rectal tumors, located in the distal segment of the colorectal tract, often exhibit a more heterogeneous molecular phenotype, predominantly comprising CMS2-4 subtypes. These subtypes are associated with a tumor microenvironment that is more immunosuppressive or less inflammatory [41,51]. Additionally, the rectal microbiota tends to be more stable and less involved in systemic inflammatory processes, which aligns with the lower levels of hematological inflammatory markers observed in rectal cancer patients in both our data and the specialized literature.

In addition to microbial differences, several anatomical, embryological, and immunological factors likely contribute to the heightened systemic inflammatory state observed in CC. Anatomically, the colon has a longer length, greater surface area, and slower transit time compared to the rectum, resulting in prolonged exposure to microbial products and potential carcinogens, which can chronically stimulate mucosal immune responses and inflammation [41,52].

These embryologic and molecular distinctions are accompanied by differing expression of immunologic markers and inflammatory pathways, leading to variable recruitment of innate immune cells and cytokine production across tumor sites [53]. Collectively, these factors contribute to a more active and systemic inflammatory profile in CC compared to RC, supporting our findings and reinforcing the need for location-specific interpretation of inflammatory biomarkers in CRC patients.

#### Study Limitations

Although our study provides valuable insights into differences in systemic inflammatory status between CC and rectal cancer RC, certain methodological limitations must be acknowledged. The retrospective design limited our ability to control for relevant confounding variables such as smoking, the presence of obesity, diabetes, genetic syndromes, and concomitant medication, all of which can influence inflammatory marker levels and, consequently, clinical outcomes. Moreover, the accuracy of tumor staging—particularly the T and M components—may have been affected by variability in imaging techniques and interpretation, even when standardized protocols were followed, introducing potential classification bias. In addition, our study relied on static measurements of inflammatory markers without longitudinal follow-up, which may limit our understanding of the dynamics of therapeutic response and disease progression. A further limitation of this study is the relatively small sample size in certain subgroups—particularly T1–T2 stages, and low Charlson index—which may reduce the statistical power to detect meaningful differences. Additionally, multiple inflammatory markers were compared across several clinical variables without formal correction for multiple testing. As such, these subgroup analyses were considered exploratory, and their results should be interpreted with appropriate caution.

Nevertheless, it is important to emphasize that our study is among the few that directly compare systemic inflammatory profiles between colon and rectal cancer in a substantial patient cohort. This comparison highlights fundamental biological and clinical differences between these two entities, providing a solid basis for personalized interpretation of inflammatory biomarkers and optimization of tailored clinical management. Therefore, our findings contribute significantly to the current literature and support the need for prospective studies aimed at elucidating underlying mechanisms and validating the prognostic utility of these biomarkers as disease-specific tools.

## 5. Conclusions

Our study demonstrates clear and significant differences in systemic inflammatory profiles between colon cancer and rectal cancer, with a markedly enhanced inflammatory response in colon cancer, especially in advanced T stages, and in patients undergoing elective surgery. Although survival outcomes show no statistical difference between CC and RC, these findings highlight the necessity for nuanced, tumor site-specific interpretation of inflammatory biomarkers to optimize personalized management strategies in colorectal cancer. By elucidating the biological heterogeneity between colon and rectal tumors, our work advances understanding in the field and provides a framework for future research aimed at refining prognostic tools and tailoring therapeutic approaches.

## Figures and Tables

**Table 1:** Key characteristics of the investigated cohorts.

Characteristic	All, *n* = 296	Colon Cancer, *n* = 148	Rectal Cancer, *n* = 148	*p*
Age (M ± SD), years	62.43 ± 8.79	63.07 ± 8.38	61.80 ± 9.20	0.445
Gender, men	190 (64.2%)	94 (63.5%)	96 (64.9%)	0.904
Rural	123 (41.6%)	48 (32.4%)	75 (50.7%)	0.002
CHARLSON > 3	162 (54.9%)	70 (47.3%)	92 (62.6%)	0.010
Emergency	78 (26.4%)	34 (23%)	44 (29.7%)	0.235
Relapse	29 (9.8%)	6 (4.1%)	23 (15.5%)	0.001
Stage				1
I	28 (9.5%)	14 (9.5%)	14 (9.5%)	
II	84 (28.4%)	42 (28.4%)	42 (28.4%)	
III	152 (51.4%)	76 (51.4%)	76 (51.4%)	
IV	32 (10.8%)	16 (10.8%)	16 (10.8%)	
Lymphatic invasion	123 (42.1%)	82 (55.4%)	41 (27.7%)	<0.001
pT				0.002
1	6 (2%)	4 (2.7%)	2 (1.4%)	
2	32 (10.8%)	12 (8.1%)	20 (13.5%)	
3	181 (61.1%)	80 (54.1%)	101 (68.2%)	
4	77 (26%)	52 (35.1%)	25 (16.9%)	
pN				0.489
0	116 (39.2%)	56 (37.8%)	60 (40.5%)	
1	114 (38.5%)	58 (39.2%)	56 (37.8%)	
2	66 (22.3%)	34 (23%)	32 (21.7)	
pM	32 (10.8%)	16 (10.8%)	16 (10.8%)	1

M = mean, SD = standard deviation.

**Table 2:** Variation of inflammatory markers CC vs. RC.

Marker	All, *n* = 296	Colon Cancer, *n* = 148	Rectal Cancer, *n* = 148	*p*
Lymphocytes	1850 ± 768	1697 ± 681	2048 ± 830	<0.001
Monocytes	651 ± 215	655 ± 124	647 ± 252	0.950
Platelets	313,100 ± 126,912	331,581 ± 137,239	289,242 ± 108,195	0.007
Neutrophils	5670 ± 2717	6094 ± 3161	5285 ± 2185	0.034
NLR	3.39 ± 2.19	3.99 ± 2.2	2.84 ± 1.53	<0.001
MLR	0.4 ± 0.23	0.43 ± 0.18	0.35 ± 0.19	0.245
PLR	195.15 ± 115.22	219.84 ± 129.27	163.28 ± 84.47	<0.001
AISI	1126.37 ± 907.76	1714.68 ± 1108.26	593.55 ± 458.9	0.009
SIRI	2.76 ± 2.18	3.7 ± 3.76	1.91 ± 1.4	0.004
SII	1173.85 ± 1284.8	1533.83 ± 1284.9	847.83 ± 629.31	<0.001

**Table 3:** Variation of inflammatory markers in cases with Charlson < 3. CC vs. RC.

Marker	CC, *n* = 78	RC, *n* = 55	*p*
Lymphocytes	1665 ± 711	2055 ± 892	0.022
Monocytes	756 ± 170	647 ± 185	0.590
Neutrophils	5944 ± 3596	5554 ± 2349	0.555
Platelets	332,997 ± 151,989	338,589 ± 116,530	0.828
NLR	4.08 ± 3.06	3.2 ± 2.12	0.127
PLR	230.99 ± 141.66	196.68 ± 108.94	0.159
MLR	0.53 ± 0.45	0.38 ± 0.25	0.310
AISI	1370.98 ± 785.54	769.17 ± 685.54	0.068
SII	1405.54 ± 1084.45	1084.54 ± 845.45	0.128
SIRI	3.87 ± 1.93	2.21 ± 1.97	0.068

**Table 4:** Variation of inflammatory markers in cases with a Charlson score > 3. CC vs. RC.

Marker	CC, *n* = 71	RC, *n* = 92	*p*
Lymphocytes	1730 ± 652	2045 ± 806	0.012
Monocytes	547 ± 323	646 ± 285	0.058
Neutrophils	6232 ± 2731	5004 ± 1771	0.007
Platelets	330,041 ± 120,263	262,151 ± 94,242	<0.001
NLR	3.91 ± 2.17	2.58 ± 0.92	<0.001
PLR	208.37 ± 115.06	145.14 ± 61.27	<0.001
MLR	0.34 ± 0.23	0.33 ± 0.15	0.797
AISI	1100.89 ± 773.14	487.38 ± 348.89	0.001
SII	1468.16 ± 1099.75	703.87 ± 410.67	<0.001
SIRI	2.89 ± 2.26	1.7 ± 0.88	0.001

**Table 5:** Inflammation ratios in emergency surgery. CC vs. RC.

Marker	CC, *n* = 34	RC, *n* = 44	*p*
Lymphocytes	1650± 684	1628 ± 780	0.907
Monocytes	476 ± 314	620 ± 298	0.065
Neutrophils	6.41 ± 3.94	5.80 ± 3.41	0.303
Platelets	320,625 ± 150,987	304,000 ± 103,749	0.610
NLR	4.50 ± 2.95	3.94 ± 2.38	0.490
PLR	211.13 ± 126.59	215.48 ± 100.73	0.881
AISI	1166 ± 815	917.50 ± 777.59	0.315
SII	1652.06 ± 1013	1226 ± 937.82	0.246
SIRI	3.16 ± 2.75	2.79 ± 2.30	0.624

**Table 6:** Inflammation ratios in elective surgery. CC vs. RC.

Marker	CC (M ± SD) *n* = 114	RC (M ± SD) *n* = 104	*p*
Lymphocytes	1710 ± 682	2221 ± 791	<0.001
Monocytes	705 ± 140	658 ± 231	0.726
Neutrophils	5.87 ± 2.91	5.1 ± 1.5	0.041
Platelets	334,769 ± 133,548	283,187 ± 110,046	0.004
NLR	3.85 ± 2.53	2.44 ±0.78	<0.001
PLR	222.17 ± 130.44	141.86 ± 66.47	<0.001
MLR	0.47 ± 0.19	0.31 ± 0.08	0.077
AISI	1606.29 ± 527.99	577.26 ± 322.55	0.013
SII	1350.09 ± 509.21	711.99 ± 401.11	<0.001
SIRI	3.84 ± 2.32	1.59 ± 0.68	0.003

**Table 7:** Inflammation ratios in T1–T2 early tumors. CC vs. RC.

Marker	CC, *n* = 16	RC, *n* = 22	*p*
Lymphocytes	1810 ± 964	2467 ± 885	0.044
Monocytes	577 ± 276	754 ± 197	0.041
Neutrophils	4352 ± 1634	5765 ± 276	0.022
Platelets	245,125 ± 71,687	336,600 ± 148,520	0.022
NLR	2.63 ± 1.47	2.43 ±1.47	0.627
PLR	189.92 ± 154.41	139.94 ± 41.95	0.143
MLR	0.45 ± 0.19	0.36 ± 0.05	0.296
AISI	551.19 ± 474.46	617.98 ± 349.45	0.702
SII	711.71 ± 520.49	815.59 ± 444.47	0.536
SIRI	1.94 ± 1.23	1.77 ± 0.33	0.720

**Table 8:** Inflammation ratios in T3–T4 advanced tumors. CC vs. RC.

Marker	CC, *n* = 132	RC, *n* = 126	*p*
Lymphocytes	1683 ± 640	1956 ± 792	0.006
Monocytes	664 ± 131	623 ± 257	0.733
Neutrophils	6391 ± 3268	5176± 2276	0.006
Platelets	342,560 ± 139,823	278,718 ± 94,931	<0.001
NLR	4.22 ± 2.71	2.94 ±1.67	<0.001
PLR	222.24 ± 126.25	168.46 ± 90.62	<0.001
MLR	0.43 ± 0.12	0.36 ± 0.21	0.349
AISI	1713.33 ± 741	587.87 ± 498.66	0.008
SII	1474.2 ± 763.49	855.33 ± 6666.9	<0.001
SIRI	4 ± 2.14	1.94 ± 1.55	0.004

## Data Availability

The original contributions presented in the study are included in the article, further inquiries can be directed to the corresponding author.

## References

[1] Abreu Lopez B.A., Pinto-Colmenarez R., Caliwag F.M.C., Ponce-Lujan L., Fermin M.D., Cortés A.V.G., Martínez A.G.M., Martinez I.G.Z., León F.G. (2024). Colorectal Cancer Screening and Management in Low- and Middle-Income Countries and High-Income Countries: A Narrative Review. Cureus.

[2] International Agency for Research on Cancer (2024). Data Version: Globocan 2022, Version 1.1.

[3] Ola I., Cardoso R., Hoffmeister M., Brenner H. (2024). Utilization of colorectal cancer screening tests: A systematic review and time trend analysis of nationally representative data. eClinicalMedicine.

[4] Ola I., Cardoso R., Hoffmeister M., Brenner H. (2025). Recent trends in self-reported utilization of colonoscopy and fecal occult blood test in Europe: Analysis of the European Health Interview Surveys 2013–2015 and 2018–2020. Eur. J. Epidemiol..

[5] Wang C.C., Sung W.W., Yan P.Y., Ko P.Y., Tsai M.C. (2021). Favorable colorectal cancer mortality-to-incidence ratios in countries with high expenditures on health and development index: A study based on GLOBOCAN database. Medicine.

[6] Barna R., Dema A., Jurescu A., Văduva A.O., Lăzureanu D.-C., Vița O., Natarâș B., Hurmuz I., Vidac A., Tăban S. (2025). The Relevance of Sex and Age as Non-Modifiable Risk Factors in Relation to Clinical-Pathological Parameters in Colorectal Cancer. Life.

[7] Leijssen L.G.J., Dinaux A.M., Kunitake H., Bordeianou L.G., Berger D.L. (2018). Pathologic factors are more important than tumor location in long-term survival in colon cancer. Int. J. Color. Dis..

[8] Gheju A., Jurescu A., Tăban S., Al-Jobory D., Lazăr F., Dema A. (2021). Different disease characteristics in young patients with colorectal cancer: A large retrospective study in a city in Romania. J. Int. Med. Res..

[9] Alinia S., Ahmadi S., Mohammadi Z., Shirvandeh F.R., Asghari-Jafarabadi M., Mahmoudi L., Safari M., Roshanaei G. (2024). Exploring the impact of stage and tumor site on colorectal cancer survival: Bayesian survival modeling. Sci. Rep..

[10] Brouwer N.P.M., Bos A.C.R.K., Lemmens V.E.P.P., Tanis P.J., Hugen N., Nagtegaal I.D., de Wilt J.H., Verhoeven R.H. (2018). An overview of 25 years of incidence, treatment and outcome of colorectal cancer patients. Int. J. Cancer.

[11] Kim M., Son I.T., Oh B.Y. (2023). Inflammatory Response Markers as Predictors of Colorectal Cancer Prognosis. Ewha Med. J..

[12] Yamamoto T., Kawada K., Obama K. (2021). Inflammation-Related Biomarkers for the Prediction of Prognosis in Colorectal Cancer Patients. Int. J. Mol. Sci..

[13] Yamamoto T., Fukuda M., Okuchi Y., Oshimo Y., Nishikawa Y., Hisano K., Kawai T., Iguchi K., Okuda Y., Kamimura R. (2022). Clinical impact of lymphocyte/C-reactive protein ratio on postoperative outcomes in patients with rectal cancer who underwent curative resection. Sci. Rep..

[14] Wang J., Liu Y., Jiang W., Zhang D., Cheng C., Liu C., Zhao Z., Wang H. (2025). Predictive value of the systemic inflammation grade for overall survival in patients with colorectal cancer after surgery: Outperforming NLR and mGPS. Front. Oncol..

[15] Ma L., Yang F., Guo W., Tang S., Ling Y. (2024). Prognostic role of platelet-to-lymphocyte ratio in patients with rectal cancer undergoing resection: A systematic review and meta-analysis. Front. Oncol..

[16] Moise G.V., Feier C.V.I., Gaborean V., Faur A.M., Rus V.I., Muntean C. (2025). From Blood to Outcome: Inflammatory Biomarkers in Rectal Cancer Surgery at a Romanian Tertiary Hospital. Diseases.

[17] Ciocan A., Ciocan R.A., Al Hajjar N., Gherman C.D., Bolboacă S.D. (2021). Abilities of Pre-Treatment Inflammation Ratios as Classification or Prediction Models for Patients with Colorectal Cancer. Diagnostics.

[18] Menyhart O., Fekete J.T., Győrffy B. (2024). Inflammation and Colorectal Cancer: A Meta-Analysis of the Prognostic Significance of the Systemic Immune-Inflammation Index (SII) and the Systemic Inflammation Response Index (SIRI). Int. J. Mol. Sci..

[19] Yang Y., Hu Z., Ye Y., Wu H., Sun W., Wang N. (2025). Association of aggregate index of systemic inflammation with increased all-cause and cardiovascular mortality in female cancer patients. Front. Oncol..

[20] Șerban R.E., Popescu D.M., Boldeanu M.V., Florescu D.N., Șerbănescu M.-S., Șandru V., Panaitescu-Damian A., Forțofoiu D., Șerban R.-C., Gherghina F.-L. (2025). The Diagnostic and Prognostic Role of Inflammatory Markers, Including the New Cumulative Inflammatory Index (IIC) and Mean Corpuscular Volume/Lymphocyte (MCVL), in Colorectal Adenocarcinoma. Cancers.

[21] Ose J., Gigic B., Hardikar S., Lin T., Himbert C., Warby C.A., Peoples A.R., Lindley C.L., Boehm J., Schrotz-King P. (2022). Presurgery Adhesion Molecules and Angiogenesis Biomarkers Are Differently Associated with Outcomes in Colon and Rectal Cancer: Results from the ColoCare Study. Cancer Epidemiol. Biomark. Prev..

[22] Lino-Silva L.S., Salcedo-Hernández R.A., Ruiz-García E.B., García-Pérez L., Herrera-Gómez Á. (2016). Pre-operative Neutrophils/Lymphocyte Ratio in Rectal Cancer Patients with Preoperative Chemoradiotherapy. Med. Arch..

[23] Absenger G., Szkandera J., Stotz M., Postlmayr U., Pichler M., Ress A.L., Schaberl-Moser R., Loibner H., Samonigg H., Gerger A. (2013). Preoperative neutrophil-to-lymphocyte ratio predicts clinical outcome in patients with stage II and III colon cancer. Anticancer Res..

[24] Andor M., Man D.E., Nistor D.C., Buda V., Dragan S. (2024). The Influence of COVID-19 in Glycemic Control: Predictive Value of Inflammation and Metabolic Parameters. Biomedicines.

[25] Daliu P., Bogdan I., Rosca O., Licker M., Stanga L.C., Hogea E., Vaduva D.B., Muntean D. (2025). Fungal Pulmonary Coinfections in COVID-19: Microbiological Assessment, Inflammatory Profiles, and Clinical Outcomes. Biomedicines.

[26] Fericean R.M., Rosca O., Citu C., Manolescu D., Bloanca V., Toma A.-O., Boeriu E., Dumitru C., Ravulapalli M., Barbos V. (2022). COVID-19 Clinical Features and Outcomes in Elderly Patients during Six Pandemic Waves. J. Clin. Med..

[27] Zhang Y., Liu X., Xu M., Chen K., Li S., Guan G. (2020). Prognostic value of pretreatment systemic inflammatory markers in patients with locally advanced rectal cancer following neoadjuvant chemoradiotherapy. Sci. Rep..

[28] Bouvier A.M., Jooste V., Lillini R., Marcos-Gragera R., Katalinic A., Rossi P.G., Launoy G., Bouvier V., Guevara M., Ardanaz E. (2024). Differences in survival and recurrence of colorectal cancer by stage across population-based European registries. Int. J. Cancer.

[29] Sepassi A., Li M., Zell J.A., Chan A., Saunders I.M., Mukamel D.B. (2024). Rural-Urban Disparities in Colorectal Cancer Screening, Diagnosis, Treatment, and Survivorship Care: A Systematic Review and Meta-Analysis. Oncologist.

[30] Moss J.L., Wang M., Liang M., Kameni A., Stoltzfus K.C., Onega T. (2021). County-level characteristics associated with incidence, late-stage incidence, and mortality from screenable cancers. Cancer Epidemiol..

[31] Morishima T., Matsumoto Y., Koeda N., Shimada H., Maruhama T., Matsuki D., Nakata K., Ito Y., Tabuchi T., Miyashiro I. (2019). Impact of Comorbidities on Survival in Gastric, Colorectal, and Lung Cancer Patients. J. Epidemiol..

[32] Ostenfeld E.B., Nørgaard M., Thomsen R.W., Iversen L.H., Jacobsen J.B., Søgaard M. (2013). Comorbidity and survival of Danish patients with colon and rectal cancer from 2000–2011: A population-based cohort study. Clin. Epidemiol..

[33] Ouchi A., Komori K., Kinoshita T., Oshiro T., Kunitomo A., Ito S., Abe T., Shimizu Y. (2021). Possibilities for and limits of upfront surgical strategy with lateral pelvic node dissection for low rectal cancer. Jpn. J. Clin. Oncol..

[34] Basile D., Garattini S.K., Corvaja C., Montico M., Cortiula F., Pelizzari G., Gerratana L., Audisio M., Lisanti C., Fanotto V. (2020). The MIMIC Study: Prognostic Role and Cutoff Definition of Monocyte-to-Lymphocyte Ratio and Lactate Dehydrogenase Levels in Metastatic Colorectal Cancer. Oncologist.

[35] Coussens L.M., Werb Z. (2002). Inflammation and cancer. Nature.

[36] Chen J.H., Zhai E.T., Yuan Y.J., Wu K.-M., Xu J.-B., Peng J.-J., Chen C.-Q., He Y.-L., Cai S.-R. (2017). Systemic immune-inflammation index for predicting prognosis of colorectal cancer. World J. Gastroenterol..

[37] Xie Q.K., Chen P., Hu W.M., Sun P., He W.-Z., Jiang C., Kong P.-F., Liu S.-S., Chen H.-T., Yang Y.-Z. (2018). The systemic immune-inflammation index is an independent predictor of survival for metastatic colorectal cancer and its association with the lymphocytic response to the tumor. J. Transl. Med..

[38] Moro-Valdezate D., Martín-Arévalo J., Cózar-Lozano C., García-Botello S., Pérez-Santiago L., Casado-Rodrigo D., Martínez-Ciarpaglini C., Tarazona N., Pla-Martí V. (2025). Prognostic value of routine blood biomarkers in 3-year survival of resectable colorectal cancer patients: A prognostic nomogram for clinical practice. Int. J. Color. Dis..

[39] Tominaga T., Nonaka T., Oyama S., Takamura Y., Hashimoto S., Shiraishi T., Sawai T., Nagayasu T. (2023). Efficacy of Neutrophil-to-Lymphocyte Ratio for Cancer-Specific Survival in Elderly Patients with Localized Colon Cancer: A Single Center Propensity Score-Matched Analysis. Clin. Exp. Gastroenterol..

[40] Guinney J., Dienstmann R., Wang X., de Reyniès A., Schlicker A., Soneson C., Marisa L., Roepman P., Nyamundanda G., Angelino P. (2015). The consensus molecular subtypes of colorectal cancer. Nat. Med..

[41] Nagtegaal I.D., Odze R.D., Klimstra D., Paradis V., Rugge M., Schirmacher P., Washington K.M., Carneiro F., Cree I.A., The WHO Classification of Tumours Editorial Board (2020). The 2019 WHO classification of tumours of the digestive system. Histopathology.

[42] Mlecnik B., Bindea G., Angell H.K., Maby P., Angelova M., Tougeron D., Church S.E., Lafontaine L., Fischer M., Fredriksen T. (2016). Integrative Analyses of Colorectal Cancer Show Immunoscore Is a Stronger Predictor of Patient Survival Than Microsatellite Instability. Immunity.

[43] Grivennikov S.I., Greten F.R., Karin M. (2010). Immunity, inflammation, and cancer. Cell.

[44] Hernandez-Ainsa M., Velamazan R., Lanas A., Carrera-Lasfuentes P., Piazuelo E. (2022). Blood-Cell-Based Inflammatory Markers as a Useful Tool for Early Diagnosis in Colorectal Cancer. Front. Med..

[45] An S., Shim H., Kim K., Kim B., Bang H.-J., Do H., Lee H.-R., Kim Y. (2022). Pretreatment inflammatory markers predicting treatment outcomes in colorectal cancer. Ann. Coloproctol..

[46] Dolan R.D., McSorley S.T., Park J.H., Watt D.G., Roxburgh C.S., Horgan P.G., McMillan D.C. (2018). The prognostic value of systemic inflammation in patients undergoing surgery for colon cancer: Comparison of composite ratios and cumulative scores. Br. J. Cancer.

[47] Kostic A.D., Chun E., Robertson L., Glickman J.N., Gallini C.A., Michaud M., Clancy T.E., Chung D.C., Lochhead P., Hold G.L. (2013). *Fusobacterium nucleatum* potentiates intestinal tumorigenesis and modulates the tumor-immune microenvironment. Cell Host Microbe.

[48] Chen T., Li Q., Wu J., Wu Y., Peng W., Li H., Wang J., Tang X., Peng Y., Fu X. (2018). *Fusobacterium nucleatum* promotes M2 polarization of macrophages in the microenvironment of colorectal tumours via a TLR4-dependent mechanism. Cancer Immunol. Immunother..

[49] Rubinstein M.R., Wang X., Liu W., Hao Y., Cai G., Han Y.W. (2013). *Fusobacterium nucleatum* promotes colorectal carcinogenesis by modulating E-cadherin/β-catenin signaling via its FadA adhesin. Cell Host Microbe.

[50] Bullman S., Pedamallu C.S., Sicinska E., Clancy T.E., Zhang X., Cai D., Neuberg D., Huang K., Guevara F., Nelson T. (2017). Analysis of Fusobacterium persistence and antibiotic response in colorectal cancer. Science.

[51] Galasso L., Termite F., Mignini I., Esposto G., Borriello R., Vitale F., Nicoletti A., Paratore M., Ainora M.E., Gasbarrini A. (2025). Unraveling the Role of *Fusobacterium nucleatum* in Colorectal Cancer: Molecular Mechanisms and Pathogenic Insights. Cancers.

[52] Dekker E., Tanis P.J., Vleugels J.L.A., Kasi P.M., Wallace M.B. (2019). Colorectal cancer. Lancet.

[53] Sarandria N. (2022). A Literature Review in Immuno-Oncology: Pathophysiological and Clinical Features of Colorectal Cancer and the Role of the Doctor-Patient Interaction. J. Cancer Ther..

